# Supplementary material for: Identification of photoperiod-induced specific miRNAs in the adrenal glands of Sunite sheep (Ovis aries)
Source: Front Vet Sci. 2022 Jul 22;9:888207. doi: 10.3389/fvets.2022.888207 (PMC9354845; doi:10.3389/fvets.2022.888207)
Supplement: Supplementary file 1 [file Data_Sheet_1.ZIP › Supplementary file/miRDeep2 file.zip!/miRDeep2 file/Novel_Structure/LP42A2/Novel_64.pdf]

Provisional ID	: NC_019459.2_3957
Score total	: 18
Score for star read(s)	: 3.9
Score for read counts	: 10.8
Score for mfe	: 1.6
Score for randfold	: 1.6
Score for cons. seed	:
Total read count	: 33
Mature read count	: 31
Loop read count	: 0
Star read count	: 2

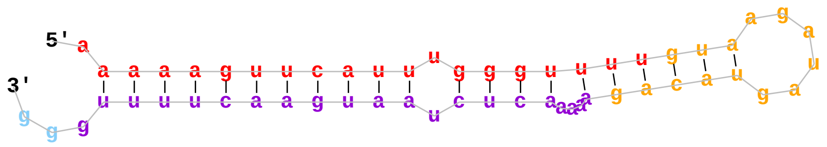

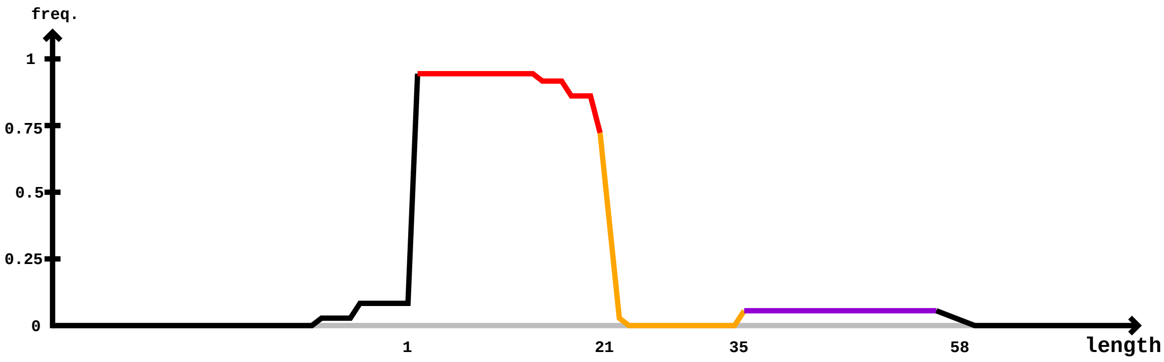


```
                                   Mature                           Star
5'- gggcugaauaauauuccacuauauaguggguuggccaaaaaguucauuuggguuuuguaagauaguacagaaaaacucuaaugaacuuuuggggcaacccaauguauuuau   -3'    obs
    gggcugaauaauauuccacuauauaguggguuggccaaaaaguucauuuggguuuuguaagauaguacagaaaaacucuaaugaacuuuuggggcaacccaauguauuuau          exp
    (((...........)))...(((((.(((((((.((.(((((((((((.((((((((((......))))))...)))).))))))))))).)).))))))).)))))....    reads    mm    sample
    ..........................uggguuggccaaaaaguucauuA..............................................................    1        1     seq
    ..............................uuggGcaaaaaguucauuuggg...........................................................    2        1     seq
    ....................................aaaaaguucGuuuggguuu........................................................    5        1     seq
    ....................................aaaaaguucGuuuggguuuu.......................................................    24       1     seq
    ....................................aaaaaguucGuuuggguuuug......................................................    1        1     seq
    ....................................aaaaaguucauuuggguuuuCu.....................................................    1        1     seq
    ......................................................................aaaaacucuaaugaacuuuug....................    2        0     seq
```
